# Relationship between malnutrition and the presence of symptoms of anxiety and depression in hospitalized cancer patients

**DOI:** 10.1007/s00520-021-06532-y

**Published:** 2021-09-21

**Authors:** Francisco José Sánchez-Torralvo, Victoria Contreras-Bolívar, María Ruiz-Vico, José Abuín-Fernández, Inmaculada González-Almendros, Manuel Barrios, Gabriel Olveira

**Affiliations:** 1 Unidad de Gestión Clínica de Endocrinología y Nutrición, Hospital Regional Universitario de Málaga, Málaga, Spain; 2 Instituto de Investigación Biomédica de Málaga (IBIMA), Málaga, Spain; 3 Universidad de Málaga, Málaga, Spain; 4 Unidad de Gestión Clínica de Endocrinología Y Nutrición, Hospital Universitario San Cecilio, Granada, Spain; 5 Unidad de Gestión Clínica de Oncología Médica, Hospital Regional Universitario de Málaga, Málaga, Spain; 6 Unidad de Gestión Clínica de Radiodiagnóstico, Hospital Regional Universitario de Málaga, Málaga, Spain; 7 Unidad de Gestión Clínica de Hematología y Hemoterapia, Hospital Regional Universitario de Málaga, Málaga, Spain

**Keywords:** Cancer, Oncology, Anxiety, Depression, HADS, Malnutrition, GLIM criteria

## Abstract

**Background:**

Anxiety and depression are a common issue in patients with cancer, yet understudied among hospitalized patients. The aim of this study was to estimate the prevalence of anxiety and depression symptomatology in cancer inpatients and its relationship with malnutrition.

**Methods:**

Cross-sectional study in hospitalized cancer patients. A nutritional assessment was done using the Global Leadership Initiative on Malnutrition (GLIM) criteria to diagnose malnutrition. Data regarding anxiety and depression symptomatology was obtained with the Hospital Anxiety and Depression Scale (HADS).

**Results:**

A total of 282 inpatients were assessed. GLIM criteria found 20% (66) of well-nourished and 80% (216) with malnutrition. HADS presented an average score of 8.3 ± 4.4 with respect to anxiety and an average score of 7.7 ± 4.6 with respect to depression. Up to 54% of the patients showed a possible presence of anxiety, and 45.3% of them showed a possible presence of depression. In malnourished patients, HADS score was non-significantly higher with respect to anxiety (8.5 ± 4.3 in malnourished vs 7.1 ± 4.6 in well-nourished; *p* = 0.06) and was significantly higher with respect to depression (8.2 ± 4.6 in malnourished vs 5.3 ± 4.0 in well-nourished; *p* < 0.001). After controlling for potential confounders, malnourished patients were 1.98 times more likely to present anxious symptomatology (95% CI 1.01–3.98; *p* = 0.049) and 6.29 times more likely to present depressive symptomatology (95% CI 1.73–20.47; *p* = 0.005).

**Conclusions:**

The presence of anxiety and depression symptomatology in oncological inpatients is high. There is an association between malnutrition and presenting anxious and depressive symptomatology in hospitalized cancer patients.

## Introduction

Cancer disease and nutritional status are closely related, as symptoms caused by the disease, the associated secondary complications, and the antineoplastic therapies increase the risk of an impaired nutritional status [1, 2]. Malnutrition, defined as “a state resulting from lack of intake or uptake of nutrition that leads to altered body composition and body cell mass leading to diminished physical and mental function and impaired clinical outcome from disease” [3], is a common issue among patients with cancer, being present in up to 80% of them and increasing morbidity and mortality [4]. The prevalence of malnutrition increases in advanced-stage cancer patients who require hospital admission [5].

In cancer patients, the presence of psychological problems such as depression and anxiety can make it difficult to manage and control the disease [6]. Previous studies have reported that the prevalence of depression among cancer patients is two to three times higher than in the general population [7]. Depression and anxiety occur up to 20% and 10% of patients with cancer, respectively [8], although the figures vary depending on factors such as the stage of the disease or the phase of treatment [8, 9]. Thus, some authors estimate a higher prevalence, especially in patients with advanced cancer or palliative therapy [10, 11].

Depression is associated with poor adherence to cancer treatment and poor cancer survival [12]. Depression and anxiety disorder are associated with an increased risk of mortality and their co-occurrence further increased the risk [13]. Cancer patients with anxiety and depression were at greater risk for emergency department visits and hospitalizations, experienced longer hospital stays, and accrued higher healthcare costs [14].

The relationship between malnutrition and psychological distress has been described in cancer patients. Patients experiencing weight loss and other symptoms of malnutrition often report higher levels of psychological distress, which manifest as more severe fatigue, insomnia, anxiety, and depression, further contributing to disease progression [15]. The symptoms of malnutrition and psychological distress overlap, but few studies have focused on the relationship between them, being depression and anxiety the problems most frequently endorsed by patients as contributing to its appearance [16]. With regard to depression specifically, there are studies that indicate its relationship with less food consumption, weight loss, and malnutrition [16, 17].

As far as we know, published data on the prevalence of symptoms of anxiety and depression at its relationship with malnutrition in hospitalized cancer patients are scarce [18]. These patients also tend to have more advanced stages, which could condition this possible association.

Our hypothesis is that the prevalence of anxiety and depression symptoms is high in hospitalized cancer patients and is related to the high rate of malnutrition in these patients [5].

The aim of the study was to assess the relationship between malnutrition and the level of anxiety and depression in hospitalized patients with cancer.

## Methods

**Fig. 1:**
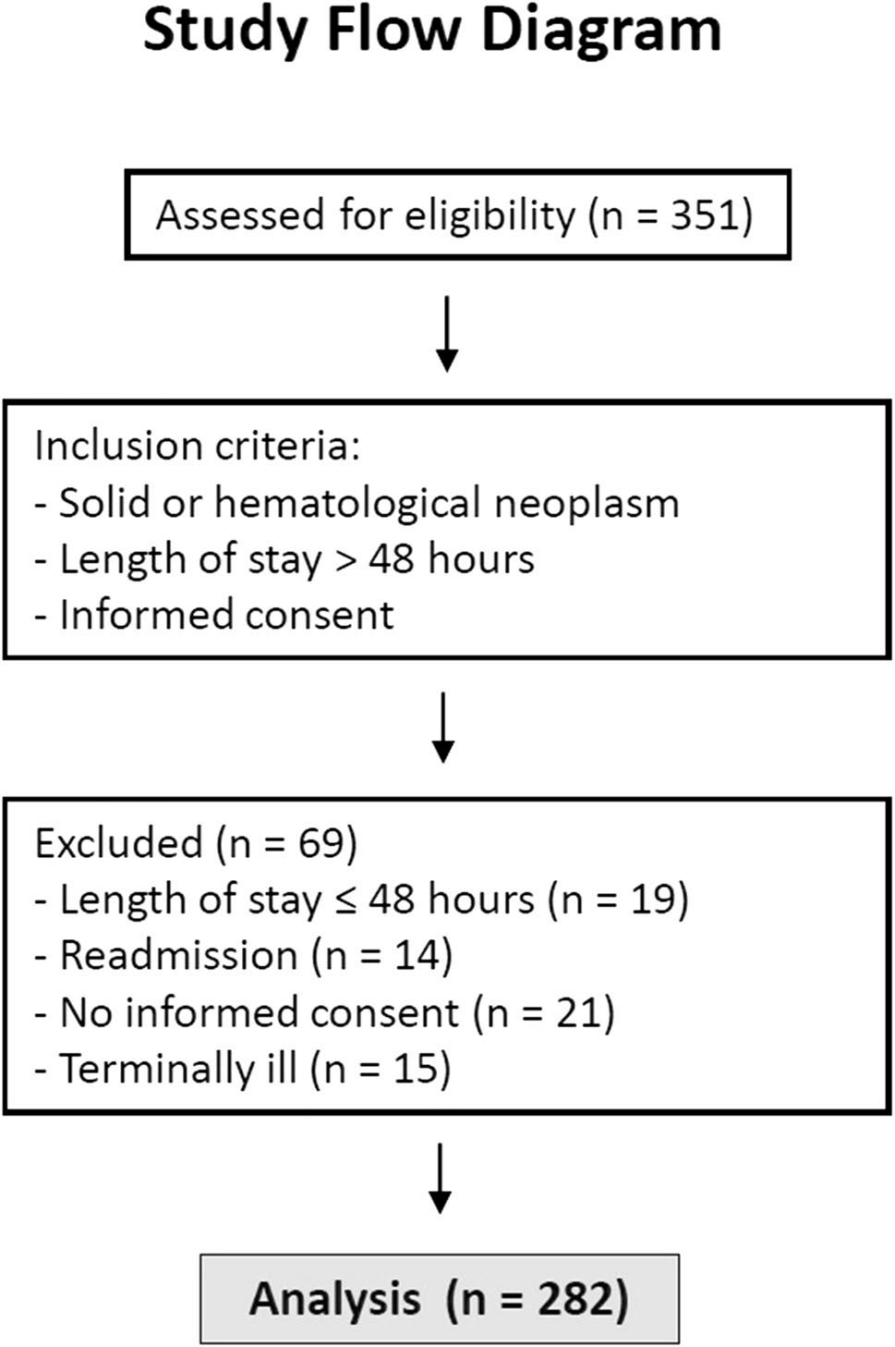
Study flow diagram

This was an observational, prospective study of clinical practice performed at Hospital Regional Universitario de Málaga. A total of 351 patients were assessed for eligibility after admission to the oncology ward. Inclusion criteria were patients with solid or hematological neoplasm, length of stay above 48 h, and able to sign the informed consent. Exclusion criteria were patients with length of stay below 48 h, readmission before 1 month, and a situation of actively dying. After screening of inclusion/exclusion criteria, 282 patients were included and 69 were excluded (Fig. 1).

### Assessment of malnutrition

We performed a nutritional assessment according to GLIM criteria within the first 24 h after admission. To diagnose malnutrition, at least one phenotypic criterion and one etiologic criterion should be present [19].

#### Phenotypic criteria

We assessed unintentional weight loss (> 5% in 6 months), low BMI (for age < 70 years normal values were considered as BMI ≥ 20 kg/m^2^, and for age ≥ 70 normal values were established as BMI ≥ 22 kg/m^2^), and/or reduction of muscle mass basing hand grip strength (HGS). Weight was assessed with a weighing scale adjusted to 0.1 kg (SECA 665, Germany) and height was obtained by a stadiometer (Holtain Limited, Crymuch, UK). With these two values, body mass index (BMI) was calculated.

Handgrip strength was used to estimate muscle mass [5] and was measured in the dominant hand with a Jamar dynamometer *(Asimow Engineering Co., Los Angeles, CA*). The patients performed the test while sitting with shoulder adducted and forearm neutrally rotated, elbow flexed to 90º, and forearm and wrist in neutral position. Patients were told to perform three consecutive contractions 1 min apart from each other, and mean value was calculated. Results were expressed in absolute figures and compared with the reference population [20]. Values under the fifth percentile were considered as low strength.

#### Etiologic criteria

Etiologic criteria were assessed by reduced food intake (estimated as per quartiles) or assimilation (as per clinical record), and/or inflammatory response of the disease (patients were considered as presenting chronic disease-related inflammation using Glasgow prognostic score) [19, 21].

### The questionnaire

The presence of symptoms of anxiety and depression was measured with Hospital Anxiety and Depression Scale (HADS) questionnaire. The HADS is a brief patient self-report measure of anxiety and depression requiring patients to report their symptoms during the previous week [22]. The HADS has demonstrated reliability and validity in cancer patient populations [23] and has been found to be an effective screening tool for cancer patients currently undergoing treatment [24].

The HADS consists of 14 items and two subscales, one measuring anxiety (HADS Anxiety subscale), with seven items, and another measuring depression (HADS Depression subscale), with seven items, which score separately.

HADS Anxiety subscale focus mainly on symptoms of generalized anxiety disorder and HADS Depression subscale is focused on anhedonia, the main symptom of depression. Each item is scored on a response-scale with four alternatives ranging between 0 and 3. The possible scores range from 0 to 21 for each of the two subscales, taking 2–5 min to complete [22]. Subscale scores from 0 to 7 are classified as normal. Subscale scores ranging from 8 to 11 are typically used for identifying the possible presence of anxiety and depression (“doubtful cases”) and subscale scores over 11 indicates the probable presence (“caseness”) of a mood disorder [25].

### Data analysis

Quantitative variables were expressed as the mean ± standard deviation. Quantitative variables distribution was assessed using Kolmogorov–Smirnov test. Differences between quantitative variables were analyzed using Student’s *t* test and, for variables not following a normal distribution, using non-parametric tests (Mann–Whitney or Kruskal–Wallis). We designed multivariate logistic regression models in which the dependent variable was the cut-off points of the HADS subscales according to malnutrition determined by GLIM criteria, controlling also for sex, age, and tumor stage. For calculations, significance was set at *p* < 0.05 for two tails. The data analysis was performed with the SPSS 22.0 program (SPSS Inc., Chicago, IL, 2013).

## Results

A total of 282 patients admitted to inpatient oncology unit were evaluated. Mean age was 60.4 ± 12.6 years, 55.7% were male. Their general features are displayed in Table 1. Most patients (92.9%) had an advanced-stage tumor (17.7% stage III, 75.2% stage IV). The most frequent types of neoplasm were lung (25.2%), colon (13.0%), breast (13%), and esophagogastric (11.8%). At the moment of admission, a nutritional assessment according to GLIM criteria was performed, detecting 80% (216) of patients with malnutrition (Table 1). Furthermore, only 20.6% (58) of the patients had a low BMI and 37.9% (107) had a low HGS, which could be related to a high prevalence of sarcopenic obesity.

**Table 1:** General features. Prevalence of anxiety and depression symptomatology

		*n* = 282
Age (years)	Mean ± SD	60.4 ± 12.6
Sex	*n* (%)	
Men		157 (55.7)
Women		125 (44.3)
Tumor stage	*n* (%)	
I		7 (2.5)
II		13 (4.6)
III		50 (17.7)
IV		212 (75.2)
BMI (kg/m^2^)	Mean ± SD	
Men		24.7 ± 4.9
Women		24.5 ± 5.1
Hand-grip strength (kg)	Mean ± SD	
Men		26.21 ± 8.58
Women		16.51 ± 6.70
Malnutrition according to GLIM criteria	*n* (%)	216 (80%)
HADSA score	Mean ± SD	8.30 ± 4.40
HADSD score	Mean ± SD	7.68 ± 4.61
Possible presence of anxiety (HADSA ≥ 8)	*n* (%)	127 (54%)
Possible presence of depression (HADSD ≥ 8)	*n* (%)	106 (45.3%)
Probable presence of anxiety (HADSA ≥ 11)	*n* (%)	62 (26.4%)
Probable presence of depression (HADSD ≥ 11)	*n* (%)	63 (26.9%)

*BMI* body mass index; *GLIM* Global Leadership Initiative on Malnutrition; *HADSA* Hospital Anxiety and Depression Scale Anxiety subscale; *HADSD* Hospital Anxiety and Depression Scale Depression subscale; *SD* standard deviation

HADS presented an average score of 8.3 ± 4.4 points with respect to anxiety (8.0 ± 4.5 points in male vs 8.7 ± 4.3 points in female) and an average score of 7.7 ± 4.6 with respect to depression (7.5 ± 4.8 points in male vs 7.8 ± 4.3 points in female). With these data, 54% of our patients showed a possible presence of anxiety and 45.3% showed a possible presence of depression.

**Fig. 2:**
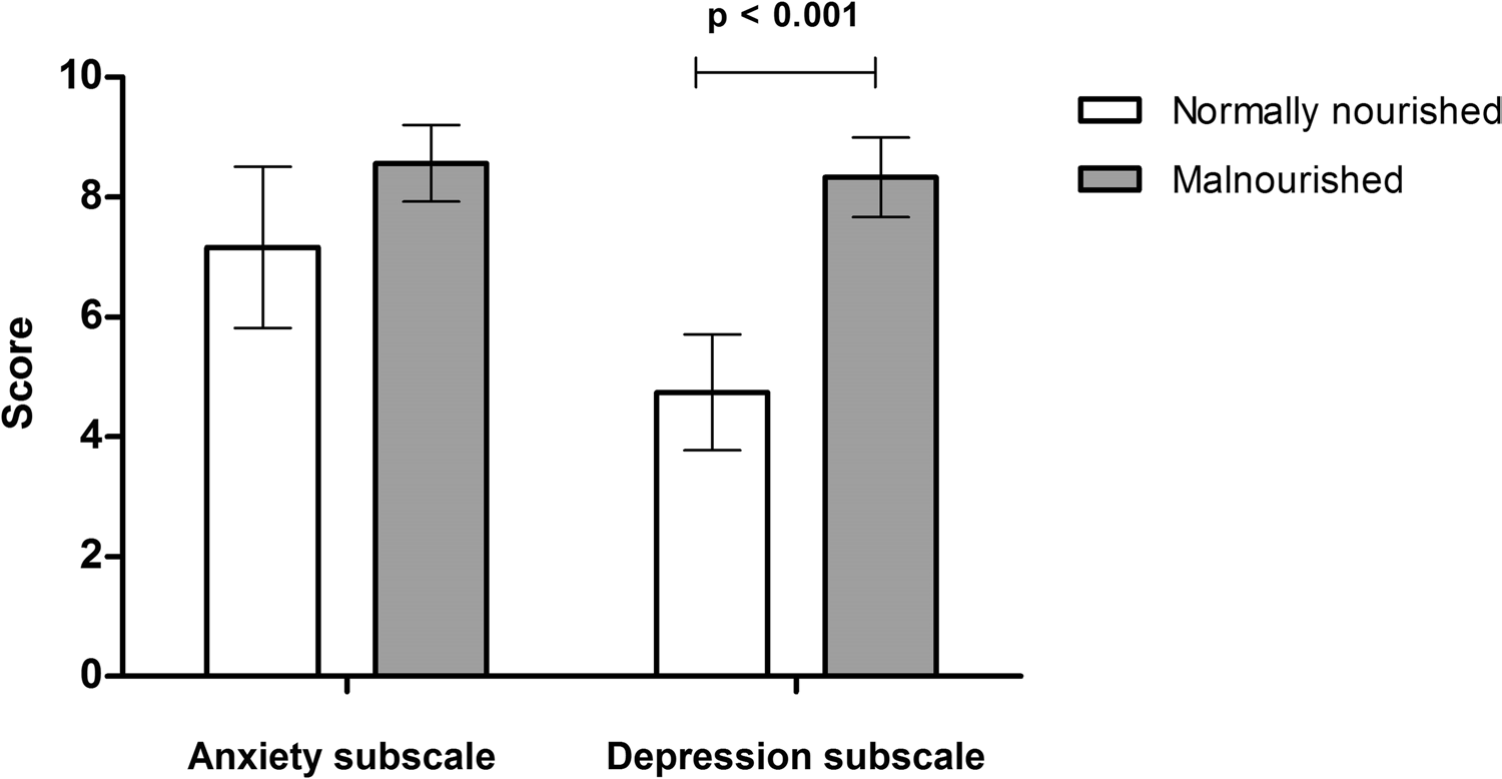
Association between malnutrition and HADS Anxiety and Depression subscales

In malnourished patients according to GLIM criteria, average score was non-significantly higher with respect to anxiety (8.5 ± 4.3 points in malnourished vs 7.1 ± 4.6 in well-nourished; *p* = 0.06) and was significantly higher with respect to depression (8.2 ± 4.6 points in malnourished vs 5.3 ± 4.0 points in well-nourished; *p* < 0.001) (Fig. 2).

Table 2 shows the logistic regression data (crude and adjusted) for the risk of presenting anxious or depression symptomatology in malnourished patients. After controlling for potential confounders, in malnourished patients according to GLIM criteria, the odds ratio of presenting anxious symptomatology was 1.98 times greater than in well-nourished [95% CI 0.99–3.98; *p* = 0.05] and the odds ratio of presenting depression symptomatology in these patients was 6.29 times greater than in well-nourished [95% CI 1.73–20.47; *p* = 0.005].

**Table 2:** Risk of presenting anxious or depression symptomatology in malnourished patients. Adjusted for age, sex, and cancer stage

	Crude				Adjusted			
	Odds ratio	95% CI		*P* value	Odds ratio	95% CI		*P* value
		Lower	Upper			Lower	Upper	
Possible presence of anxiety (HADSA ≥ 8)	2.06	1.04	4.06	**0.037**	1.98	1.01	3.98	**0.049**
Probable presence of anxiety (HADSA ≥ 11)	1.03	0.48	2.21	0.93	1.05	0.48	2.32	0.89
Possible presence of depression (HADSD ≥ 8)	6.73	2.71	16.73	** < 0.001**	6.29	2.51	15.75	** < 0.001**
Probable presence of depression (HADSD ≥ 11)	6.08	1.81	20.48	**0.004**	5.95	1.73	20.47	**0.005**

Bold: statistically significant

## Discussion

The presence of anxiety and depression symptomatology in cancer inpatients is high, and there is an association between this symptomatology and malnutrition in our study.

We used HADS questionnaire to identify anxiety and depressive symptoms. Cancer research has extensively applied subscale thresholds of 11 to indicate the likely presence of anxiety and/or depression, reported as achieving 70–95% sensitivity and 83% specificity with respect to clinical interview [26] although some authors suggest that the recommended cut-off scores for the HADS may result in under-reporting of psychiatric morbidity among patients with cancer [27]. An optimal balance between sensitivity and specificity was found using a cut-off score of 8 or above for both HADS Anxiety and HADS Depression [26].

Previous studies in cancer patients found a prevalence of 12–15% of anxiety symptomatology and a prevalence of 5.7–7% of depression symptomatology using HADS threshold of ≥ 11 [28]. In the present study, using a HADS threshold of ≥ 11, 26.9% of patients were identified with a likely presence of depression and 24.4% of them were identified with a probable presence of anxiety. Using a HADS threshold of ≥ 8, highlights that close to half of respondents were identified with a likely presence of depression and 54% of them were identified with a probable presence of anxiety.

Even using a HADS threshold of ≥ 11, the rate of anxiety and depression symptomatology in our series is higher than in the series previously described.

Most of the patients of our study (92.9%) had an advanced-stage tumor (17.7% stage III, 75.2% stage IV), many of them under palliative therapy. Prevalence of anxiety and depression is high in advanced stages of the disease. Světláková et al. found that the baseline prevalence of anxiety and depression symptomatology in 126 patients treated with palliative systemic therapy for advanced cancer was 35.9 and 56.5%, respectively [11].

Figures also vary by cancer type, with depression affecting an estimated 17.9% of patients with lung cancer, 16.5% of those with gynecological cancers, 15.6% in breast cancer, 13.2% in genitourinary cancers, and 10.7% in gastrointestinal cancers [7]. The highest levels of anxiety are reported in gynecological, lung, head, and neck and breast cancers [7]. The most frequent types of neoplasm in our series were lung (25.2%) and breast (13%), which could explain higher rates of anxious and depressive symptoms.

A review and meta-analysis showed the prevalence of major depression (15%), minor depression (20%), and anxiety (10%) in patients treated for cancer [8].

Linden et al. performed a study with 10,153 cancer outpatients using the Psychosocial Screen for Cancer questionnaire, a cancer-specific screening instrument for anxiety and depression that was validated using the HADS questionnaire [29]. Across cancer types, 19.0% of patients showed clinical levels of anxiety and another 22.6% had subclinical symptoms. Further, 12.9% of patients reported clinical symptoms of depression and an additional 16.5% described subclinical symptoms [7].

Brintzenhofe-Szoc et al. performed a study in a 8235 adult outpatients using the Brief Symptom Inventory questionnaire (BSI-18), a widely used screening measure in cancer patients that contains three subscales: somatization, anxiety, and depression [30]. Mixed anxiety/depression symptoms were seen in 12.4% of patients; depression symptoms in 18.3%, and anxiety symptoms in 24.0% [31]. A former study conducted in Spain [10], which included outpatients who had undergone cancer surgery in the previous month and who initiated adjuvant chemotherapy, showed a prevalence of malnutrition of 36.4%, depression symptoms in 35.5%, and anxiety symptoms in 35.2% of the patients using also the BSI-18.

We found an association between presenting anxious and depression symptomatology and malnutrition. Previous studies also point towards this association [18, 32–36]. Chabowski et al. found that malnutrition is associated with a significant worsening in terms of depression, anxiety, and pain. [18]. Gosak et al. found a significant correlation between malnutrition and HADS scores, with more distressed patients found in the malnourished/cachectic subgroups [33]. Santos et al. found the presence of depression in 52.9% elderly malnourished patients undergoing treatment for cancer [37]. The utility of a short depression screening in predicting malnutrition has been demonstrated [35].

The link between malnutrition and major depression is both complicated and correlative. It remains unclear whether depression in cancer patients is the cause or consequence of impaired nutritional status [38]. Van Liew et al. demonstrated that there is a direct reciprocal association between depressive symptoms and weight loss: changes in either variable were associated with concurrent changes in the other variable [36]. Therefore, depression might lead to a lack of appetite, loss of interest in self-care, apathy, and physical weakness. These conditions appear to be linked in a cycle whereby major depression might cause anorexia and result in malnutrition, whereas malnutrition related with cancer directly impair appetite [34]. Biological mechanisms linking distress and malnutrition have been identified, suggesting a link between cancer-related anorexia and depression caused by an impairment of serotonin [16].

The presence of malnutrition and poor recognition of depression and anxiety is strongly associated with reduced quality of life and survival in cancer patients [10, 12, 13]. However, given that some risk factors for nutritional decline in cancer patients are non-modifiable (e.g., stage, site, treatment), it is noteworthy that psychological symptoms are treatable, which may lead to an improvement in nutritional status [39]. However, 73% of these depressed cancer patients do not receive effective psychiatric treatment, and only 5% see a mental health professional [6]. These observations underline the importance of the assessment and treatment of both psychological symptoms and nutritional assessment.

Our study has several strengths; it includes a sufficient sample size to achieve adequate statistical power. Furthermore, it includes techniques that are already routinely taken, making it easily applicable to a clinical setting.

All the same, there are potential limitations in our study. It was a single-center cross-sectional study; thus, results should be interpreted with caution. It could only provide clues for a causality relationship between these two factors, without ruling out reverse causation. Besides, our study lacks a screening for anxiety, depression or eating disorders prior to cancer diagnosis, which could have yielded more accurate figures for the incidence of these conditions.

The high rates of anxiety and depression symptomatology found in our study, and the strong relationship found between malnutrition and psychological distress are a robust argument for implementation of systematic screening for psychological distress, and for comprehensive psychosocial support for all patients with advanced cancer throughout the disease trajectory. In turn, our findings also reinforce the need for correct malnutrition screening and nutritional counseling and support in hospitalized cancer patients.

In conclusion, the presence of anxiety and depression symptomatology in oncological inpatients is high. There is an association between malnutrition and presenting anxious and depression symptomatology in hospitalized oncologic patients. The significant levels of depression and anxiety in these patients indicate the need for early supportive psychotherapy or pharmacological interventions. Further studies are needed to better understand the causality of this association.

## Data Availability

The data that support the findings of this study are available from the corresponding author upon reasonable request.
